# Evaluation of a portable fluorometer for the quantification of vitamin E in blood at key physiological stages of dairy cattle

**DOI:** 10.3168/jdsc.2023-0520

**Published:** 2024-02-01

**Authors:** Eric J. Owczarzak, Nick Grotenrath, Hannah Carlson, Laman Mamedova, Barry J. Bradford, Angel Abuelo

**Affiliations:** 1 Department of Large Animal Clinical Sciences, College of Veterinary Medicine, Michigan State University, East Lansing, MI 48824; 2 Department of Animal Science, College of Agriculture and Natural Resources, Michigan State University, East Lansing, MI 48824

## Abstract

Vitamin E is essential in mitigating the impact of oxidative stress on periparturient dairy cows and neonatal calves. Therefore, it is essential to measure circulating vitamin E concentrations accurately. Currently, the only reliable method is an expensive and time-consuming procedure using liquid chromatography-mass spectrometry (LC-MS). However, a cheaper and faster method has been developed, which allows the quantification of circulating vitamin E through the use of a handheld fluorometric analyzer (HFA) called the vitamin E iCheck (BioAnalyt GmbH). Our objective was to compare the accuracy of the HFA to the reference LC-MS method for measuring vitamin E in bovine samples. A total of 177 samples collected for other studies were used: 98 newborn calf serum samples from a vitamin E supplementation study (including treated and control animals) and 79 whole-blood samples from cows 1 to 7 d postcalving. Vitamin E concentrations were measured on thawed calf serum and fresh cow EDTA blood using the HFA, following the manufacturer's instructions. Whole blood from cows was then centrifuged to obtain plasma. Vitamin E was also quantified in calf serum and cow plasma at the Michigan State University Veterinary Diagnostic Laboratory using LC-MS. Calf and cow results were analyzed separately because they represent different biological matrices and physiological times. In each dataset, results between the HFA and LC-MS determinations were compared using Passing-Bablok regressions and Bland-Altman plots. The HFA showed a poor linear relationship with LC-MS for calf serum and cow plasma (intercept = 0.33 and 0.67 μg/mL, respectively). The HFA unreliably estimated vitamin E, with a mean bias of −3.2 and 0.6 μg/mL for calves (vitamin E concentration range: 0.28 to 30.75 μg/mL) and cows (0.8 to 5.88 μg/mL), respectively. Moreover, 40.4% of the calf samples read below the linear range of acceptable results for the HFA, making it unsuitable for this age group. Hence, under the conditions of our study, the HFA yielded unreliable results and cannot be recommended for field use.

## Highlights

• The HFA underestimated calf serum vitamin E and had moderate correlation to LC-MS.
• The HFA overestimated cow blood vitamin E and had poor correlation to LC-MS.
• Of the calf samples, 40.4% had readings below the HFA acceptable linear range.
• The HFA yielded unreliable results and cannot be recommended for field use.

## Graphical Abstract

**Figure:**
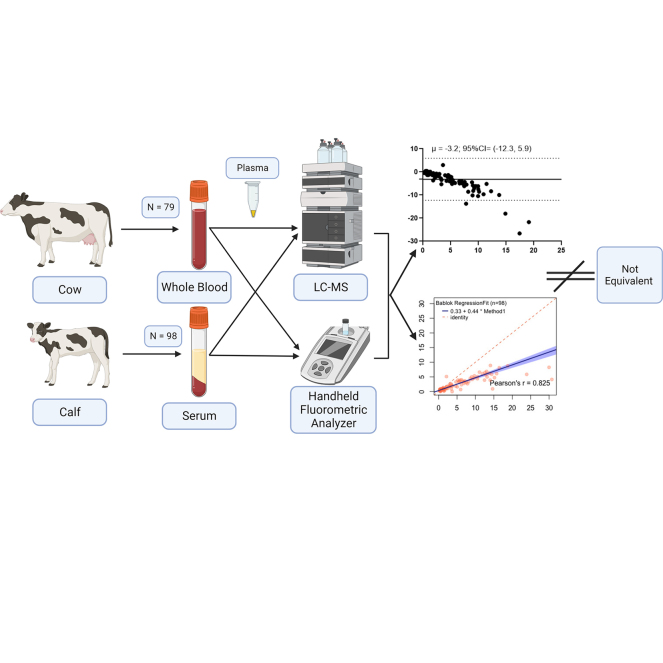
**Summary:** Circulating vitamin E concentrations have been measured using the liquid chromatography-mass spectrometry (LC-MS) reference method and an alternate method using a handheld fluorometric analyzer (HFA). A total of 98 serum samples were collected from 33 calves as part of a supplementation study and were analyzed by the HFA and LC-MS. Also, 79 whole-blood samples were collected from cows 1 to 7 days postcalving and analyzed by the HFA, with the remaining blood centrifuged to collect plasma and subsequently analyzed by LC-MS. After analysis using both Bland-Altman plots and Passing-Bablok regression, it was found that the HFA was not suitable for determining circulating vitamin E concentrations compared with LC-MS under the conditions of our study.

In the productive cycle of dairy cows, the periparturient and neonatal periods are the times of greatest disease susceptibility (Mellor and Stafford, 2004; LeBlanc et al., 2006). Oxidative stress (**OS**), the oxidative damage resulting from an imbalance between pro-oxidant production and antioxidant availability, is an underlying factor for this increased disease risk (Abuelo et al., 2019). Substantial evidence indicates that adult dairy cows and neonatal calves experience OS around the time of calving (Sordillo and Aitken, 2009; Abuelo et al., 2013; 2015a) and during the first few weeks of life (Gaál et al., 2006; Abuelo et al., 2014; Ranade et al., 2014).

Antioxidants are essential to mitigate the impact of OS on cattle health (Hogan et al., 1993). The antioxidant vitamin E protects against reactive oxygen species-mediated damage (Traber and Atkinson, 2007). However, circulating vitamin E concentrations are low during the periparturient (Weiss et al., 1997) and neonatal (Herdt and Stowe, 1991; NRC, 2001; Lashkari et al., 2021) periods of cattle, thus potentially contributing to the increased disease risk observed at these times. Further, supplementation with vitamin E during the periparturient period has been associated with a lower incidence of infectious diseases (Weiss et al., 1994, 1997; NRC, 2001). This positive relationship between vitamin E status and health outcomes has resulted in the widespread supplementation of dairy cows and calves with vitamin E (Abuelo et al., 2015b). However, excessive vitamin E supplementation can also result in adverse health events, such as an increased risk of mastitis (Bouwstra et al., 2010a,b).

The ability to accurately measure circulating vitamin E concentrations at these stages is, therefore, crucial for developing and monitoring dairy cattle supplementation strategies. Liquid chromatography-mass spectrometry (**LC-MS**) is the reference method for quantifying vitamin E in serum or plasma (Lauridsen et al., 2001; Mottier et al., 2002). However, this requires sample submission to an external specialized laboratory, increasing turnaround time and cost. At the Michigan State University Veterinary Diagnostic Laboratory (**MSU-VDL**; East Lansing, MI), plasma and serum samples can be processed and analyzed for vitamin E concentrations using their routine LC-MS method for $31 per sample. Recently, a cowside handheld fluorometric analyzer (**HFA**) using autofluorescence of vitamin E has been developed to quantify vitamin E in whole blood or serum within minutes at approximately a cost of $8.60 per sample after the initial investment in the device (iCheck Vitamin E; BioAnalyt GmbH). A previous study has validated this device in bovine samples (Ghaffari et al., 2019), indicating acceptable agreement between the HFA and the reference method, although with a lower correlation in calf than in cow samples. However, this study included only a limited number of cow (n = 28) and calf (n = 11) samples and did not specify the animals' physiological stage or supplementation status. Thus, evaluating the accuracy of this cowside analyzer at times when the circulating vitamin E concentrations are low (physiological stage) or high (supplementation) is essential before they can be used for monitoring cattle health, supplementation effectiveness, or in future research studies.

Our objective was to compare the accuracy of circulating vitamin E (α-tocopherol) determinations from HFA and the reference LC-MS method at both the periparturient and neonatal periods, as well as in supplemented calves. We hypothesized that the results generated from the HFA would be comparable to the LC-MS method.

To achieve the aim of this study, a convenience sample of 178 blood samples collected for other studies was used. All procedures involving animals were approved by the Michigan State University Institutional Animal Care and Use Committee before the onset of experiments (PROTO202300058 and PROTO202100242), and this committee granted an exemption to use surplus samples for this study. Calf samples were collected in a randomized clinical trial supplementing commercial injectable antioxidants at birth (Carlson et al., 2024). From this study, we randomly selected 11 calves from the same farm in each of 3 groups (saline control and 2 supplements containing vitamin E) to generate a greater variation of circulating vitamin E concentrations. Samples collected at 0 (before treatment administration), 1, and 4 wk of age were included for all 33 Holstein heifer calves for a total of 99 samples. Blood was collected via jugular vein puncture using evacuated tubes (Trace Element Serum Blood Collection Tubes, BD Vacutainer). After 30 min, tubes were centrifuged on the farm at 2,000 × *g* for 15 min at room temperature. The serum was then aliquoted into cryogen tubes (Corning), flash frozen in liquid nitrogen, and transported to the laboratory, where samples were stored at −80°C pending analysis within 4 mo of collection. One aliquot was submitted to the MSU-VDL and another aliquot was used for vitamin E HFA quantification in our laboratory.

Samples from periparturient cows were obtained from a cross-sectional study investigating associations between inflammatory status and herd performance. A total of 79 Holstein cows (parity 1–6) between 1 to 7 d postcalving from 2 commercial farms were blood sampled via coccygeal vessel venipuncture using evacuated tubes containing K_2_EDTA anticoagulant (BD Vacutainer). The whole blood was first used for HFA vitamin E quantification on the farm. The remaining blood was immediately centrifuged at 3,000 × *g* for 10 min at room temperature, the plasma was harvested, and stored −20°C pending submission to the MSU-VDL within 3 mo of collection. The LC-MS determinations in calf serum and cow plasma samples were performed simultaneously.

The concentration of α-tocopherol in the samples was determined using an HFA (iCheck; BioAnalyt) and reagents from the same manufacturer (Vitamin E test kit; BioAnalyt) that are based on autofluorescence of α-tocopherol extracted from the serum via n-hexane and alcohols (Ghaffari et al., 2019). The fluorometer was calibrated before each use with the calibration kit included with the fluorometer. For this assay, each sample was processed as follows: 500 μL of sample were pipetted into a vial containing 2 mL of extraction reagent, which was then vigorously shaken for 10 s and then allowed to sit for 5 min at room temperature. After standing for 5 min, the vitamin E was considered to be extracted into the organic phase and was ready to be measured. The vial was then inserted into the HFA, measured, and then recorded. One of the serum samples reported an error during HFA measurement and could not be re-assayed due to insufficient sample volume. Thus, only results from 98 calf serum samples were used for comparison analyses. The HFA determinations took place in the laboratory for thawed calf serum samples and on farm for fresh whole-blood samples from periparturient cows. For comparison with the reference method, the whole-blood results were adjusted to plasma values assuming a packed cell volume of 32% (Raila et al., 2012) because only serum or plasma but not whole blood can be measured via LC-MS.

At the MSU-VDL, calf serum and cow plasma vitamin concentrations were analyzed using ultrahigh-performance liquid chromatography using a previously published method (Arnaud et al., 1991). Briefly, the fat-soluble vitamins, including α-tocopherol, were extracted with hexane (Fisher Scientific). A vitamin E standard was prepared using vitamin E (Cerilliant) and 100% ethanol (Fisher Scientific) with 0.01% butylated hydroxytoluene (Sigma Aldrich). A 5-point standard curve was constructed using analytical standards for vitamin E ranging in concentrations from 0.01 to 100 μg/mL. Samples were then chromatographically separated on a Shimadzu LC30AD ultra-high-performance liquid chromatography system (Shimadzu, Kyoto Japan) using a Waters (Milford, MA) Ethylene Bridged Hybrid C18: 1.7 μm (2.1 × 50 mm) column. Identification and quantification of peaks was done using an ABSciex 6500+ triple quadrupole mass spectrometer (ABSciex, Framingham, MA). The peaks were reviewed manually by trained MSU-VDL personnel.

The reference LC-MS and HFA methods were compared following the American Society of Veterinary Clinical Pathology guidelines (Jensen and Kjelgaard-Hansen, 2006). Data were analyzed using Bland-Altman plots (GraphPad Prism) and regression analyses in RStudio, using packages *tidyverse* (Wickham, 2023), *readxl* (Wickham and Bryan, 2023), *lme4* (Bates et al., 2023), and *mcr* (Potapov et al., 2023). Data from calves and cows were analyzed separately given the differences in sample matrix in the HFA assay (serum vs. whole blood). The normality of data was assessed with the Shapiro-Wilk test. Spearman correlation coefficients between concentrations obtained using the LC-MS and HFA methods were calculated. Regression analyses were used to estimate constant and proportional error via intercept and slope, respectively (Westgard and Hunt, 1973). A perfect agreement between methods would show no constant error with an intercept = 0, no proportional error with a slope = 1, and all points falling on the regression line. Bland-Altman plots were also used to visualize the difference in measurements between 2 different measuring devices which cannot normally be seen by only correlation estimates (Bland and Altman, 1986). In this plot, the horizontal solid line represents the mean difference between the methods as well as 2 dotted lines representing the 95% CI.

The calf serum and cow whole-blood concentrations determined by the HFA ranged from 0 to 8.9 μg/mL. The calf serum and cow plasma concentrations determined by LC-MS ranged from 0.28 to 30.75 μg/mL (Figure 1). The HFA manufacturer reports a linear range of acceptable results from 1 to 25 μg/mL. Thus, values falling above or below this range are not reliable. Within our calf serum samples measured by the HFA, 40.4% (n = 40/99) of samples had vitamin E readings below this linear range.

**Figure 1:**
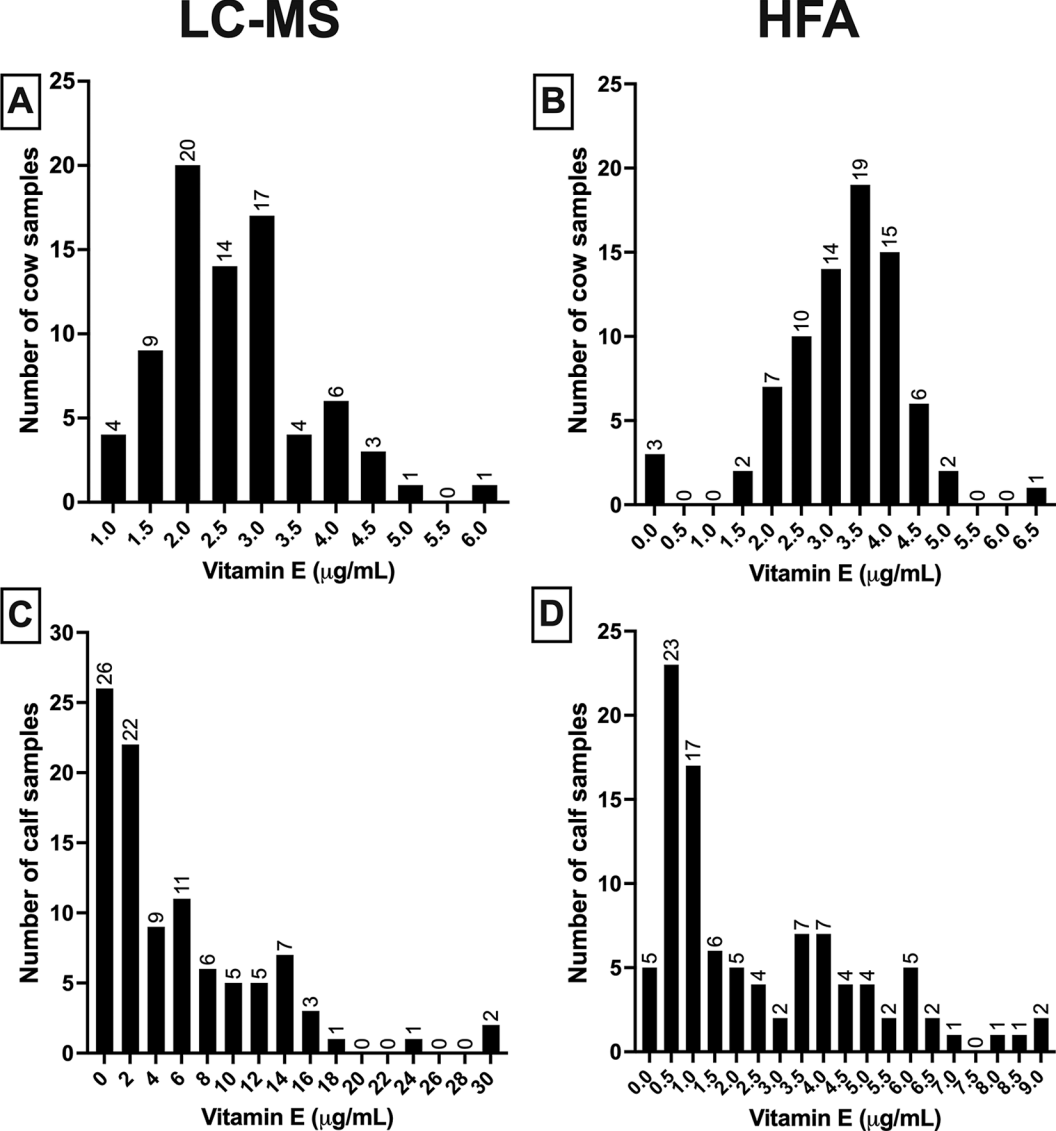
Histograms of the frequency distribution of the vitamin E concentrations analyzed with LC-MS (panels A and C) and HFA (panels B and D) in cow (panels A and B) and calf (panels C and D) samples. Bars represent the number of samples with concentrations in each histogram bin.

Spearman correlation coefficients between HFA and LC-MS determinations were ρ = 0.87 (*P* < 0.001) and ρ = 0.40 (*P* = 0.004) for calf serum and cow plasma samples, respectively, indicating just a moderate to weak association between the results of both methods. Linear regression analyses revealed a poor linear fit between both methods (R^2^ = 0.68 and 0.16 for the calf and cow samples, respectively). Linear regression assumes normality of the data distribution and absence of error in the reference method. We, therefore, subsequently used Passing-Bablok regression because this nonparametric technique has no special assumptions regarding the distribution of the data or errors (Passing and Bablok, 1983).

The Spearman rank correlation from Passing-Bablok regression (Figure 2) showed a moderate and poor correlation for the calf serum (ρ = 0.83) and cow plasma (ρ = 0.30) samples, respectively. For both the calf serum and cow plasma samples, the HFA showed an intercept larger than zero (0.33 and 0.67, respectively), indicating that the HFA systematically overestimates the concentration of circulating vitamin E compared with LC-MS. In addition, the calf serum samples presented a slope of 0.44, showing that the HFA greatly underestimated vitamin E concentration compared with LC-MS as the sample concentration increased. The cow plasma samples presented a near-perfect slope of 1.01, showing that there is a very low proportional error. However, there is still a poor correlation, indicating a large systematic error within the range of measurements. Ultimately, the combination of systemic error in both calf serum and cow plasma samples, and proportional error in the calf samples make the results of the HFA unreliable to inform the vitamin E status of the animals and potential subsequent management changes such as targeted supplementation.

**Figure 2:**
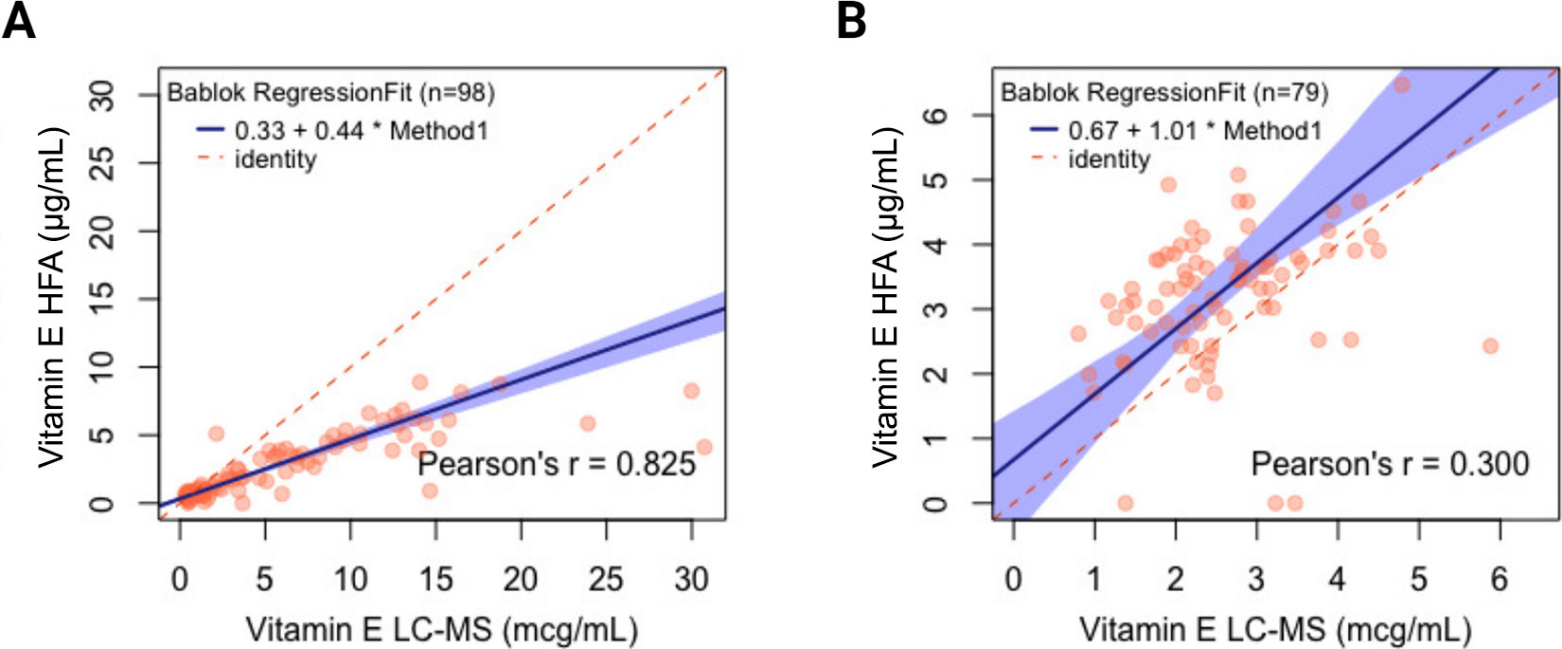
Passing-Bablok regression analysis between circulating vitamin E concentrations measured using the reference LC-MS method and HFA. A total of 98 serum samples were used from Holstein calves (A) and 79 whole-blood samples from Holstein cows between 1 and 7 d postcalving (B). The LC-MS quantification of adult cow samples occurred in plasma, and the whole-blood HFA value was corrected assuming a 32% packed cell volume. The red dashed line is the identity line (Y = X) and represents perfect agreement between both methods. The blue line represents the regression line and the blue shadowed area its 95% CI.

In the calf serum samples, the Bland-Altman analyses revealed a mean bias of −3.2 (95% CI: −12.3 to 5.9) μg/mL of the HFA compared with the LC-MS (Figure 3). This negative bias indicates that vitamin E concentrations were underestimated by the HFA, on average, by 3.2 μg/mL. The reported mean ± SE serum vitamin E concentrations for calves not receiving any supplemental vitamin E or calves receiving 500 IU/d vitamin E are 0.88 ± 0.08 and 2.89 ± 0.08 μg/mL (Reddy et al., 1987); as such, this large underestimation by the HFA makes the use of this device unreliable for measuring physiological concentrations of vitamin E in this age group, whether calves are supplemented or not. Furthermore, the 95% CI for the bias in the calf serum samples was also broad, with a large portion of the data points falling far from the mean bias line. Based on visual assessment of the Passing-Bablok regression (Figure 2A) and the Bland-Altman plot (Figure 3A), the underestimation was greater at higher LC-MS vitamin E concentrations, even though none of the calf serum samples yielded a result above the upper limit of the HFA manufacturer's reported linear range. Overall, given the great proportion of calf serum samples falling below the linear range of the HFA and the large negative bias in vitamin E concentrations detected, the HFA does not seem an appropriate method to quantify vitamin E concentrations in neonatal calves.

**Figure 3:**
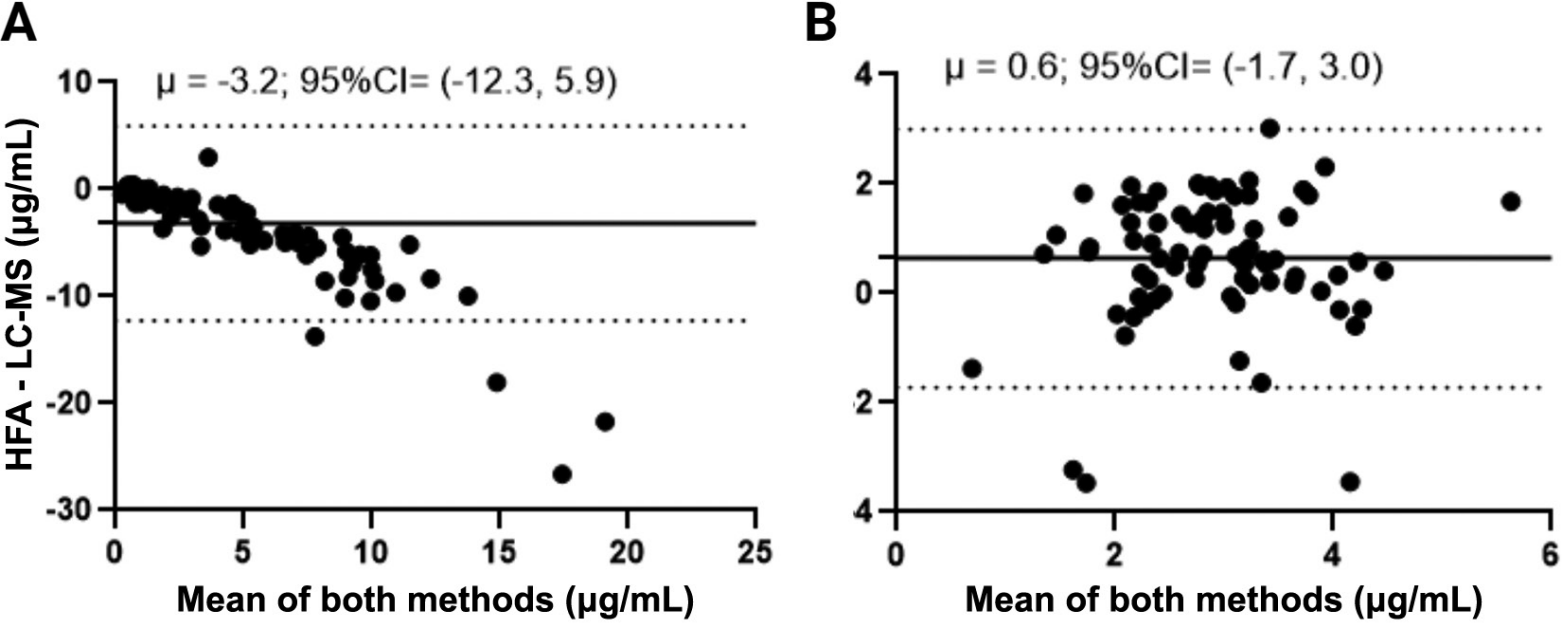
Bland-Altman plots represent the differences in circulating vitamin E concentrations measured between the reference LC-MS and HFA against the mean of both methods. A total of 98 serum samples were used from Holstein calves (A) and 79 whole-blood samples from Holstein cows between 1 and 7 d postcalving (B). The LC-MS quantification of adult cow samples occurred in plasma, and the whole-blood HFA value was corrected assuming a 32% packed cell volume. The solid black line represents the mean bias; the dashed black lines represent the 95% CI of agreement. μ = overall mean bias (μg/mL) calculated as the HFA value minus the LC-MS value.

In the cow plasma samples, the mean bias was 0.6 (95% CI: −1.7 to 3.0) μg/mL. In contrast to the calf data, vitamin E concentrations in cattle plasma samples were, on average, overestimated by the HFA. However, the CI was broad and crossed over 0, indicating that the HFA could both under- and overestimate vitamin E concentrations, which could be troubling if it were to be used to dictate supplementation needs. The magnitude of the bias is also relevant because reported mean ± SD serum vitamin E in transition cows is 2.71 ± 1.38 μg/mL (LeBlanc et al., 2004), indicating that the average serum concentration falls within the bias CI. Moreover, differences in vitamin E serum concentrations as small as 1 μg/mL are associated with changes in the risk of retained placenta (LeBlanc et al., 2004). Given the observed HFA mean bias compared with LC-MS, it is questionable whether the HFA could accurately detect concentrations in that range to inform management decisions at the farm level. Thus, our results suggest that the HFA is not an acceptable alternative for measuring circulating vitamin E concentrations in transition cows.

In this study, we used different sample matrices in calves and cows. Thus, we assessed the 2 life stages separately. Nevertheless, this resulted in the cow and calf stages being confounded by the sample matrix, not allowing us to understand if differences in performance could be due to the life stage or sample matrix. However, the HFA is marketed for both matrices used (whole blood and serum), and the overall performance of the HFA was unreliable in both groups analyzed. Another limitation of this study is that the cow whole-blood results were adjusted to plasma values using a constant packed cell volume rather than based on each cow's actual results. This could have introduced some bias in our results. However, the manufacturer also recommends an assumed packed cell volume for the final calculation of the concentration of plasma vitamin E, and this approach was used in studies validating the same or similar devices (Raila et al., 2012; Ghaffari et al., 2019). Moreover, bias due to constant packed cell volume was ruled out previously for this HFA (Ghaffari et al., 2019).

Under the conditions of our study, the HFA method is unreliable for quantifying circulating vitamin E concentrations in calves or cows. Thus, it is not recommended for the HFA to be used either at times of known physiologically low circulating vitamin E concentrations such as around calving or early in life, or to monitor vitamin E supplementation programs.
